# Machine Learning and Texture Analysis of [^18^F]FDG PET/CT Images for the Prediction of Distant Metastases in Non-Small-Cell Lung Cancer Patients

**DOI:** 10.3390/biomedicines12030472

**Published:** 2024-02-20

**Authors:** Armin Hakkak Moghadam Torbati, Sara Pellegrino, Rosa Fonti, Rocco Morra, Sabino De Placido, Silvana Del Vecchio

**Affiliations:** 1 Department of Advanced Biomedical Sciences, University of Naples “Federico II”, 80131 Naples, Italy; hakakarmin@gmail.com (A.H.M.T.); sara.pellegrino@unina.it (S.P.); rosa.fonti@unina.it (R.F.); 2 Department of Clinical Medicine and Surgery, University of Naples “Federico II”, 80131 Naples, Italy; rocco.mor4@gmail.com (R.M.); deplacid@unina.it (S.D.P.)

**Keywords:** machine learning, texture features, [^18^F]FDG PET/CT, non-small-cell lung cancer, metastases

## Abstract

The aim of our study was to predict the occurrence of distant metastases in non-small-cell lung cancer (NSCLC) patients using machine learning methods and texture analysis of ^18^F-labeled 2-deoxy-d-glucose Positron Emission Tomography/Computed Tomography {[^18^F]FDG PET/CT} images. In this retrospective and single-center study, we evaluated 79 patients with advanced NSCLC who had undergone [^18^F]FDG PET/CT scan at diagnosis before any therapy. Patients were divided into two independent training (*n* = 44) and final testing (*n* = 35) cohorts. Texture features of primary tumors and lymph node metastases were extracted from [^18^F]FDG PET/CT images using the LIFEx program. Six machine learning methods were applied to the training dataset using the entire panel of features. Dedicated selection methods were used to generate different combinations of five features. The performance of selected machine learning methods applied to the different combinations of features was determined using accuracy, the confusion matrix, receiver operating characteristic (ROC) curves, and area under the curve (AUC). A total of 104 and 78 lesions were analyzed in the training and final testing cohorts, respectively. The support vector machine (SVM) and decision tree methods showed the highest accuracy in the training cohort. Seven combinations of five features were obtained and introduced in the models and subsequently applied to the training and final testing cohorts using the SVM and decision tree. The accuracy and the AUC of the decision tree method were higher than those obtained with the SVM in the final testing cohort. The best combination of features included shape sphericity, gray level run length matrix_run length non-uniformity (GLRLM_RLNU), Total Lesion Glycolysis (TLG), Metabolic Tumor Volume (MTV), and shape compacity. The combination of these features with the decision tree method could predict the occurrence of distant metastases with an accuracy of 74.4% and an AUC of 0.63 in NSCLC patients.

## 1. Introduction

Lung cancer is recognized as the leading cause of cancer death worldwide [1]. Each year, a total of 11.4% of all new cancer cases originate from the lungs, making it the second most common type of cancer [1]. At diagnosis, about 85% of patients have non-small-cell lung cancer (NSCLC), which, at histopathological examination, includes adenocarcinoma, squamous cell carcinoma, and large cell carcinoma [2]. Following diagnosis, patients undergo a complete work-up for the accurate staging of the disease that will guide the selection of the most appropriate treatment regimen for each patient [3]. Early-stage lung cancer patients are candidates for surgery or curative radiotherapy, whereas advanced-stage lung cancer patients will receive chemotherapy, chemo-radiotherapy, targeted therapy, and immunotherapy [4,5,6]. Despite the transitory success of therapy, patients with lung cancer in stages I-III will often develop distant metastases that probably were already present at diagnosis in a subclinical phase and thus being undetectable at the staging work-up.

Lung cancer diagnosis, staging, evaluation of response to treatment, and follow up are based on multimodality imaging, including Computed Tomography (CT), Positron Emission Tomography/Computed Tomography (PET/CT), and Magnetic Resonance Imaging (MRI). The recent development of methods for the analysis of medical images based on artificial intelligence (AI) significantly improved the accuracy and efficiency of image analysis in several clinical contexts, such as lung cancer screening and diagnosis, nodule detection, molecular characterization, lung cancer staging, response to treatment, and prognosis [7,8]. In particular, AI-based algorithms including machine learning, deep learning, and radiomics have significantly enhanced clinical decision making based on individual patient’s imaging, clinical, molecular, and pathological data. Machine learning methods include several algorithms that are capable of learning from data observation, thus obtaining inferences. Deep learning methods include algorithms based on artificial neural networks composed of layers of artificial neurons capable of extracting features from data and building complex models. Radiomics is based on the extraction and quantification of subvisual image features using data characterization algorithms.

Texture analysis is an emerging radiomic tool for the evaluation and quantification of spatial signal variation in a segmented volume of medical images [9,10]. A combination of texture analysis and AI-based algorithms has been applied to evaluate tumor heterogeneity, overall and progression-free survival (OS and PFS), response to therapy, and biological and molecular characteristics of different types of tumors [11,12]. In particular, previous studies performed texture analysis in lung cancer patients and showed that several features, including the coefficient of variation, dissimilarity, coarseness, and entropy, were able to predict both PFS and OS in patients [13,14,15,16,17,18]. Other studies successfully used texture analysis and machine learning or deep learning methods to discriminate involved lymph nodes from reactive lymph nodes in lung cancer patients by analyzing and processing ^18^F-labeled 2-deoxy-d-glucose {[^18^F]FDG} PET/CT or CT alone [19,20]. Only a few studies reported the use of texture analysis to predict distant metastases in NSCLC patients and even fewer combined texture analysis and machine learning methods to identify patients at high risk of developing distant metastases [21,22,23]. In the present study, we compared six different machine learning methods and selected those that combined with texture analysis could predict the development of distant metastases in lung cancer patients, thus helping clinicians to select the appropriate treatment for patients at high risk of metastatic dissemination. To this end, we analyzed primary tumors and lymph nodes that were positive at [^18^F]FDG PET/CT scan using texture analysis and tested different machine learning methods in order to develop an effective classification model. By applying a radiomic approach, we aim to exploit all the information contained in the images to test whether they can predict the development of distant metastases.

## 2. Materials and Methods

### 2.1. Patients

We retrospectively evaluated 79 patients (54 men, 25 women) using the following inclusion criteria: histologically proven non-small-cell lung cancer; stage III and IV disease; whole-body [^18^F]FDG PET/CT scan performed at our institution before any therapy; and clinical and imaging follow up for at least 6 months. The exclusion criteria were prior lung or chest malignancy; prior chemotherapy or chest radiotherapy; no pathological diagnosis on primary lung lesion; missing imaging data for analysis; and missing clinical and imaging follow up for at least 6 months. Distant metastases were detected by [^18^F]FDG PET/CT scan, whole body contrast-enhanced CT, brain MRI, and in selected cases, MRI of different anatomical districts, and were subsequently confirmed at clinical and imaging follow up. This single institutional study was approved by the local ethics committee (Protocol No. 352/18), and all subjects signed an informed consent form.

We divided the overall population into two independent cohorts based on the time of imaging (before or after 2019). The first cohort included 44 patients and was used for training purposes. The second independent cohort included 35 patients and was used for final testing purposes. The clinical characteristics of all patients are summarized in Table 1. In the training dataset, there were 11 patients in stage III (5 IIIA, 4 IIIB, and 2 IIIC), while 33 patients were in stage IV (14 IVA and 19 IVB). There were 18 patients with adenocarcinoma, 12 with squamous cell carcinoma, 2 with large cell carcinoma, and 12 with NSCLC not otherwise specified (NOS). In the final testing cohort, there were 15 patients in stage III (7 IIIB and 8 stage IIIC), while 20 patients were in stage IV (4 IVA and 16 IVB); there were 20 patients with adenocarcinoma, 8 with squamous cell carcinoma, 1 with large cell carcinoma, and 6 with NSCLC not otherwise specified (NOS). The overall population was treated according to their stage, histology, molecular pathology, age, performance status, and comorbidities [4,5,6], as reported in Table 1.

The presence of metastases was established at the time of [^18^F]FDG PET/CT in both the training and final testing cohorts.

### 2.2. [^18^F]FDG PET/CT Study

After fasting for 8 h, patients received 370 MBq of [^18^F]FDG by intravenous injection and 60 min later underwent [^18^F]FDG PET/CT scan using an Ingenuity TF scanner (Philips Healthcare, Best, The Netherlands). Before tracer injection, blood glucose level was measured in all patients and was <120 mg/dL. The parameters of the multidetector CT scan were 120 kV, 80 mAs, 0.8 s rotation time, and a pitch of 1.5; if needed, a fully diagnostic contrast-enhanced CT was performed. For PET scan acquisition, 3-dimensional mode, 3 min per bed position, and six to eight bed positions per patient were used. An ordered subset-expectation maximization algorithm was applied for image reconstruction. A filtered back projection of CT reconstructed images (Gaussian filter with 8 mm full width half maximum) was used for the attenuation correction of PET emission data. [^18^F]FDG PET/CT co-registered images in the transaxial, sagittal, and coronal planes were preliminarily visualized with Ingenuity TF software (IntelliSpace Portal V5.0, Philips Healthcare, Best, The Netherlands).

### 2.3. [^18^F]FDG PET/CT Image Analysis

**Figure 1:**
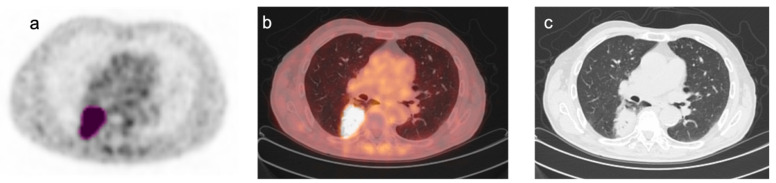
Representative images of [^18^F]FDG PET/CT scan in a patient with NSCLC. Representative images of [^18^F]FDG PET/CT scan in a patient with NSCLC: (**a**) transaxial [^18^F]FDG PET image; (**b**) co-registered PET/CT fusion image; (**c**) co-registered CT image. A tridimensional region of interest was drawn around the FDG avid tumor lesion (pink) using an automated contouring program setting an absolute threshold for SUV at 2.5 (**a**). Fusion and CT images confirmed the accuracy of lesion segmentation (**b**,**c**).

PET/CT data, after transfer in DICOM format to a different workstation, were processed by the LIFEx program [24]. Areas of focal [^18^F]FDG uptake were considered positive if detected at least on 2 contiguous PET slices and corresponded to structural CT abnormalities. When multiple lymph nodes were coalescent, they were considered as a single lesion. Areas of physiological tracer uptake were excluded from the analysis. In agreement with previous studies [25,26,27], an automated contouring program was applied for drawing a tridimensional region around the target lesion using an absolute threshold for SUV at 2.5 (Figure 1), thus obtaining a Volume of Interest (VOI). The transfer of the VOI on the corresponding CT images confirmed the accuracy of lesion delimitation.

Texture features of the primary tumor and lymph node metastases were extracted using the LIFEx package (developed at CEA, Orsay, France, http://www.lifexsoft.org, accessed on 19 February 2024). VOIs that did not reach the minimum number of 64 voxels were excluded from the analysis to avoid the inaccurate quantification of texture features inside small lesions. Tonal discretization of the gray scale for PET images was adjusted using 64 gray levels with an absolute scale bound between 0 and 25 SUV. Texture features included conventional and histogram-based parameters, shape and size, and second- and high-order features, as shown in Supplementary Table S1 online. In particular, 39 features plus conventional [^18^F]FDG PET parameters SUVmax, SUVmean, SDmean, SUVmin, SUVpeak, MTV, and TLG were extracted from each lesion and then subjected to selection based on redundancy and importance in determining the clinical endpoint.

### 2.4. Selection of the Best Machine Learning Method

There are a large number of machine learning methods that can be used to train a model. Selecting the best method highly depends on the type of data and targets. Therefore, it is often needed to apply various machine learning methods to data and compare their performance. In this study, we compared the efficacy of 6 renowned methods, including the decision tree, linear discriminant analysis, the naïve Bayes classifier, the support vector machine (SVM), k-nearest neighbors, and the feedforward neural network [28,29,30].

Briefly, the decision tree is a supervised algorithm used for classification and regression. It has a hierarchical tree-like structure composed of a root node, branches, and leaf nodes. The predicted class is obtained by applying a top-down approach to the dataset from the root node to a leaf node [31]. Linear discriminant analysis is an algorithm that finds linear discriminants that maximize the separation between classes in a dataset [32]. The naïve Bayes classifier is a supervised algorithm that computes the class of a given sample by maximizing its probability of belonging to that class. It assumes that predictors are independent of one another in each class [33]. The support vector machine is a supervised algorithm used for binary classification or regression prediction. It finds the boundary that best separates two classes while remaining at the maximum distance from the closest data points in each class. The term “support vectors” refers to the closest data points in each class [28]. K-nearest neighbor is an algorithm that identifies the k points closest to the unknown sample based on their distance. If k-nearest points belong to a given class, the assumption is that also the unknown sample belongs to the same class [34]. The feedforward neural network is a class of algorithms composed of layers of artificial neurons; basically, the input layer receives input data, the hidden layer extracts features from the input data, and the output layer produces the result. The flow of information is unidirectional from one layer to the next [35]. The selection of the tested classifiers was based on simplicity, interpretability of results, efficiency with a small dataset, computational time, and intensity. The experimental setting of each model is summarized as follows. In the decision tree model, the split criterion was the Gini’s diversity index, and the maximum tree depth was set to 10 levels. Linear discriminant analysis was performed using singular value decomposition. The naïve Bayes classifier used the Gaussian model and assumed that each class is normally distributed. The support vector machine algorithm used a linear kernel. The k-nearest neighbor algorithm used a k parameter of 1 and Euclidean distance as the distance metric. The feedforward neural network model used an input layer of 10 neurons fully connected to a hidden layer of 10 neurons followed by an output layer, using sigmoid as the activation function.

The six methods were applied to the entire set of features in the training cohort of 104 lesions using MATLAB (version R2021a, Mathworks Inc., Natick, MA, USA), and the results were subjected to univariate analysis to select the best method for the prediction of distant metastases based on accuracy. A 5-fold cross-validation approach was used to train all six methods in the training cohort of 104 lesions. Among these lesions, 70% of the input data went into the training set, leaving 30% for the validation group.

### 2.5. Selection of Texture Features

Since many features are correlated, to avoid redundancy, we calculated the Pearson correlation coefficient of pairs of features. When the correlation coefficient of the two variables was >0.8, one of the two features was removed from the analysis. To select features on the basis of their importance in determining the clinical endpoint, we applied the least absolute shrinkage and selection operator algorithm (LASSO) in a MATLAB environment along with the Cox survival model in the training cohort. This algorithm includes several selection methods, such as Fscchi2, Fscmrmr, Fscnca, Fsrftest, Fsrnca, Fsulaplacian, and Relieff, which identify a subset of measured features highly correlated with the clinical endpoint. The first 5 features selected by each method were employed in the training model.

### 2.6. Selection and Evaluation of the Best Model

The selected machine learning methods were combined with each pool of selected features and accuracy, the confusion matrix, and ROC curves were obtained to identify the best model. Then this model was used in the final testing cohort of patients. The accuracy of the model was calculated for each combination of the selected features considering the presence or absence of metastases at the time of [^18^F]FDG PET/CT.

## 3. Results

Texture analysis was performed on [^18^F]FDG PET/CT scans of NSCLC patients. A total of 104 lesions (37 primary tumors and 67 lymph nodes) were analyzed in the training group, while 78 lesions (32 primary tumors and 46 lymph nodes) were analyzed in the final testing dataset. Texture analysis provided 39 features for each lesion plus conventional [^18^F]FDG PET parameters SUVmax, SUVmean, standard deviation of SUVmean (SDmean), SUVmin, SUVpeak, Metabolic Tumor Volume (MTV), and Total Lesion Glycolysis (TLG).

Six methods were applied using the entire set of features, and univariate analysis was performed to find the best method for the prediction of metastases based on accuracy [36]. Table 2 shows the mean and standard deviation of the accuracy of each method for the training dataset. The SVM and decision tree methods had the best accuracy with 78.26% and 71.68%, respectively. So, these two methods were considered for further data processing.

Correlation among features was tested using the Pearson correlation coefficient, and 27 features (23 from texture analysis plus SUVmax, SUVmean, MTV, and TLG) remained after the exclusion of correlated features. Then, seven feature selection methods (LASSO) were applied to twenty-seven features to rank them in terms of importance and relative weight. The features extracted by each method are listed in Table 3.

The variables selected by Fscchi2 and Fsrftest and those selected by Fscnca and Fsrnca are similar since the methods have similar mathematic fundamentals. In the Fscnca and Fsrnca algorithms, the fifth feature had a slight weight compared to the previous four selected by the same method and was not included in further analysis. 

In the subsequent stage, we tested the best combination of selected features and machine learning techniques. To this end, some models were designed using features in Table 3 as the input and the presence of distant metastases as the output. These models were trained with the SVM and decision tree methods as well. Accuracy, the confusion matrix, and ROC curves were obtained to identify the best model.

Although the SVM had good accuracy in the training dataset using different combinations of features (71.4−83.7%), its accuracy was very low in the final testing dataset (40−51.3%), as shown in Supplementary Tables S2 and S3 online. For example, one of the best performances for this method belonged to a model that combined five extracted features by the Fscchi2 algorithm as inputs. Despite the fact that this model achieved fairly good accuracy in training (83.7%), the accuracy in the final testing dataset was just 51.3%. In addition, the values of the AUC derived from the ROC curve for the training and final testing datasets were very low (training AUC = 0.43, final testing AUC = 0.39).

Table 4 and Table 5 show that the decision tree method had great accuracy in both the training and final testing datasets using different combinations of features.

**Figure 2:**
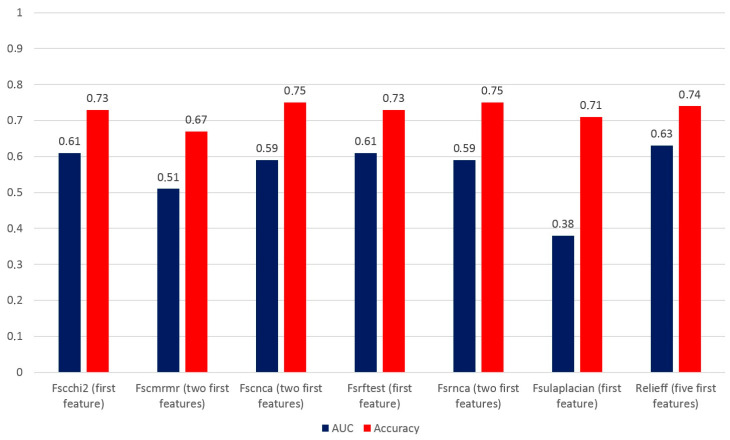
AUC and accuracy of models applied to the final testing dataset of 78 lesions using the decision tree method and different combinations of features.

Table 5 reports the accuracy of the decision tree method using different combinations of features from 1 to 5 in the final testing dataset. The best accuracy of the model using both Fscchi2 and Fsrftest was 73.1% by employing only the first feature of the combination, i.e., GLCM_dissimilarity. Using Fscnca and Fsrnca, the best accuracy was 75.5% by combining GLZLM_LZHGE with GLRLM_RLNU, the first two features of the selected combination. The best accuracy using Fscmrmr was 67.9% by combining GLRLM_RP with TLG, whereas with the Fsulaplacian combination, an accuracy of 71.8% was obtained employing only MTV. Finally, the best accuracy using Relieff was 74.4% with a combination of shape sphericity, GLRLM_RLNU, TLG, MTV, and shape compacity. To select the best model, we also consider the AUC and ROC curves, and Figure 2 shows the values of the AUC and accuracy for each model that were calculated based on the confusion matrix.

**Figure 3:**
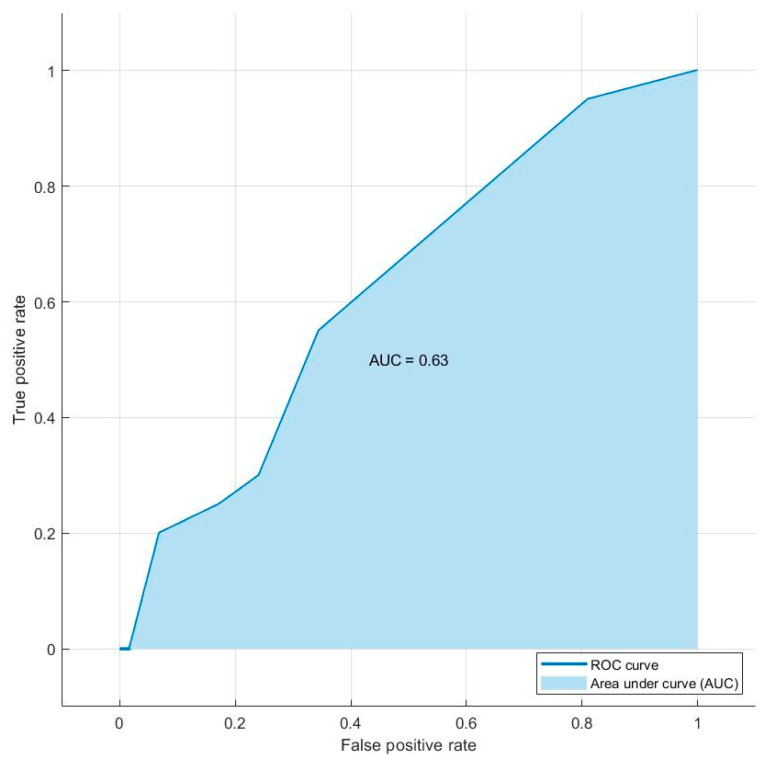
Graph of the ROC curve showing the performance of the model combining the decision tree and Relieff methods for the prediction of distant metastases in the final testing dataset of 78 lesions. The combination of shape sphericity, GLRLM_RLNU, TLG, MTV, and shape compacity is able to predict the presence of distant metastases with an AUC of 0.63.

When we took into account the AUC and accuracy of each most accurate selection method, we found that the best AUC and accuracy values were obtained using the Relieff method. Therefore, the combination of shape sphericity, GLRLM_RLNU, TLG, MTV, and shape compacity is able to predict the presence of distant metastases with an AUC of 0.63 and an accuracy of 74.4%. Figure 3 shows the AUC value and ROC curve for the combination of five features of the Relieff model.

## 4. Discussion

In the present study, we compared six machine learning methods to select the best training model for the prediction of distant metastases in NSCLC patients and found the highest accuracy using the SVM and decision tree methods on the whole set of variables. Then, by applying seven feature selection methods, we identified the best five features that were predictive of metastases. The combination of the selected machine learning methods with each group of five selected features was then tested in the training cohort of patients and evaluated in the final testing cohort. We found that by feeding the decision tree method with the combination of shape sphericity, GLRLM_RLNU, TLG, MTV, and shape compacity selected by Relieff, we were able to predict the presence of distant metastases with an AUC of 0.63 and an accuracy of 74.4% in the final testing cohort of patients. Therefore, texture analysis of [^18^F]FDG-positive lesions and the decision tree method can identify patients with a high risk of distant metastases that can be candidates for more aggressive treatments. The novelty of our study, rather than being the use of advanced AI-based algorithms, resides in the fact that a systematic evaluation of classical machine learning methods and different feature combinations even in a small cohort of patients can provide meaningful information about patient’s risk of distant metastases that can affect subsequent therapeutic choices. Another important consideration that we can draw from our results is that both GLCM_dissimilarity and MTV are very powerful predictive features since by feeding alone the decision tree model they could identify patients with distant metastases. In relation to the SVM method, while it showed good accuracy in the training group of patients using different combinations of features, it failed to predict the development of distant metastases in the final testing cohort.

Our study enrolled patients with advanced NSCLC, and most of them already had distant metastases at the time of [^18^F]FDG PET/CT examination. This allowed us to rapidly identify the features strictly correlated with the presence of distant metastases and to include them in the training model. However, the evaluation of the model in the final testing dataset, showing good accuracy, may allow us to extend the model to the early stages of NSCLC. In our study, texture analysis was performed on both primary tumors and involved lymph nodes providing a large range of feature values that reflected the heterogeneity of [^18^F]FDG uptake in lesions at different steps of disease progression. Further studies are needed to test whether combining information on the heterogeneity of primary lesions and involved lymph nodes may improve the identification of patients with early-stage NSCLC at risk of distant metastases formation.

Nevertheless, our study has several limitations, including its retrospective design, the relatively limited number of patients, a moderate cohort imbalance in relation to the percentage of stage III patients in each cohort, and the absence of external validation. Therefore, our findings may require confirmation in a larger prospective study using stringent criteria for patient enrollment, including external validation in different centers. Furthermore, the limited size of training and testing datasets prevented the application of deep learning methods. Although we are aware that these methods are powerful tools to perform even complicated tasks, we believe that a simple approach using classical machine learning methods may provide key information to build more sophisticated models with a higher clinical impact.

Previous studies used machine learning, deep learning, and radiomics for the analysis of [^18^F]FDG PET/CT images of lung cancer patients in different clinical contexts. They reported AUC values up to 0.97 in the diagnosis of lung cancer using deep learning algorithms [7,22]. In the prediction of treatment response and prognosis, AUC values up to 0.95 were achieved using machine learning algorithms [7,22]. For staging purposes, lymph node involvement was predicted by both machine learning and deep learning models, which often included radiomic features and clinical data with AUC values up to 0.94 [19,22,37,38,39,40]. Only a few studies used AI-based algorithms for the prediction of distant metastases in lung cancer patients [21,22,23]. By applying a CNN, an accuracy of 63 ± 5% was achieved in a study that included 264 patients with different stages of the disease and 102 out of 264 with distant metastases [23]. In agreement with these findings, using the decision tree method in the final testing cohort, we found an accuracy of 74.4% in the prediction of distant metastases. Deep learning was also applied to predict occult nodal metastases in [^18^F]FDG PET/CT images of patients with NSCLC [41]. In this study, 1911 patients were included in the training dataset, 355 patients for external validation and 999 patients for prospective validation. Deep learning applied to a combination of imaging features extracted from PET and CT images of primary tumors could predict occult metastases in regional lymph nodes with an AUC ranging between 0.875 and 0.958 in the different cohorts. These findings are very promising from the perspective of applying deep learning methods to the prediction of occult distant metastases in the early stages of the disease.

Therapeutic regimens in NSCLC patients are currently designed on the basis of the histology, stage, and molecular characterization of oncogene drivers and immune checkpoint proteins. The addition of features expressing the heterogeneity of glycolytic phenotypes in primary tumors and involved lymph nodes may add subclinical information on the metastatic potential of early-stage NSCLC in individual patients. Analysis of combined clinical data and texture features from imaging studies by machine learning or deep learning methods may further improve the adaptation of therapy to individual patients.

In conclusion, our study reports an effective classification model based on the machine learning method and texture analysis of [^18^F]FDG PET/CT images that can be clinically employed to identify patients with NSCLC at risk of developing distant metastases.

## Figures and Tables

**Table 1:** Clinical characteristics, histology, stage, and treatment of 79 patients with advanced NSCLC.

Characteristic	Overall	Training Cohort	Final Testing Cohort
Patients	79	44	35
Age			
Mean ± SD	65 ± 12	64 ± 13	67 ± 10
Range	38–86	38–86	41–71
Gender			
Male	54	29	25
Female	25	15	10
Histology			
Adenocarcinoma	38	18	20
Squamous cell carcinoma	20	12	8
Large cell carcinoma	3	2	1
Not otherwise specified	18	12	6
TNM stage			
Stage III	26	11	15
Stage IV	53	33	20
Treatment			
Chemotherapy	46	30	16
Chemoradiotherapy	3	3	
Chemotherapy/Immunotherapy	15	3	12
No cancer therapy	15	8	7

**Table 2:** Mean and standard deviation of the accuracy of six training machine learning methods.

Methods	Mean Accuracy (%) ± SD
Decision tree	71.68 ± 0.89
Linear discriminant analysis	65.39 ± 1.04
Naïve Bayes classification	62.05 ± 0.98
Support vector machine	78.26 ± 0.98
K-nearest neighbor	63.39 ± 1.07
Feedforward neural network	66.09 ± 1.01

**Table 3:** Features extracted by seven LASSO feature selection methods on the basis of their high correlation with the clinical endpoint.

Fscchi2	Fscmrmr	Fscnca	Fsrftest	Fsrnca	Fsulaplacian	Relieff
GLCM_ dissimilarity	GLRLM_RP	GLZLM_ LZHGE	GLCM_ dissimilarity	GLZLM_ LZHGE	MTV	Shape sphericity
GLCM_energy	TLG	GLRLM_ RLNU	GLCM_energy	GLRLM_ RLNU	SUVmean	GLRLM_ RLNU
GLCM_ homogeneity	HISTO_ kurtosis	CoV	GLCM_ homogeneity	CoV	TLG	TLG
TLG	CoV	GLRLM_ LRHGE	TLG	GLRLM_ LRHGE	CoV	MTV
GLZLM_ SZLGE	Shape sphericity		GLZLM_SZLGE		SUVmax	Shape compacity

**Table 4:** The training accuracy of the model calculated for each combination of the selected features using the decision tree method in 104 lesions.

	Number of Combined Features				
LASSO method	First feature	Two first features	Three first features	Four first features	Five first features
Fscchi2	74	75	76	82.7	79.8
Fscmrmr	76	73.1	77.9	75	74
Fscnca	77.9	78.8	72.1	71.2	-
Fsrftest	74	75	76	82.7	79.8
Fsrnca	77.9	78.8	72.1	71.2	-
Fsulaplacian	75	71.2	72.1	71.2	73.1
Relieff	76.9	76.9	76	77.9	76.9

**Table 5:** Accuracy of the model calculated for each combination of the selected features using the decision tree method in the final testing dataset of 78 lesions.

	Number of Combined Features				
LASSO method	First feature	Two first features	Three first features	Four first features	Five first features
Fscchi2	73.1	71.8	69.2	70.5	69.2
Fscmrmr	66.7	67.9	60.3	65.4	65.4
Fscnca	71.8	75.5	70.5	69.2	-
Fsrftest	73.1	71.8	69.2	70.5	69.2
Fsrnca	71.8	75.5	70.5	69.2	-
Fsulaplacian	71.8	70.1	65.4	69.5	70.6
Relieff	69.2	73.1	73.1	71.9	74.4

## Data Availability

The data presented in this study are available upon request from the corresponding author.

## Supplementary Materials

The following supporting information can be downloaded at https://www.mdpi.com/article/10.3390/biomedicines12030472/s1, Table S1: Conventional and texture features derived from [^18^F]FDG PET/CT images using LIFEx software; Table S2: Accuracy of models using the SVM method in the training dataset; Table S3: Accuracy of models using the SVM method in the final testing dataset.
