# Supplementary material for: Machine Learning and Texture Analysis of [18F]FDG PET/CT Images for the Prediction of Distant Metastases in Non-Small-Cell Lung Cancer Patients
Source: Biomedicines. 2024 Feb 20;12(3):472. doi: 10.3390/biomedicines12030472 (PMC10968471; doi:10.3390/biomedicines12030472)
Supplement: Supplementary file 1 [file biomedicines-12-00472-s001.zip › biomedicines-2841297-supplementary.pdf]

SUPPLEMENTARY INFORMATION

ARTICLE TITLE: Machine learning and texture analysis of [^{18}F]FDG PET/CT images for the prediction of distant metastases in non-small-cell lung cancer patients.

AUTHOR NAMES: Armin Hakkak Moghadam Torbati[1+], Sara Pellegrino[1+], Rosa Fonti[1], Rocco Morra[2], Sabino De Placido[2], Silvana Del Vecchio[1*].

[1]Department of Advanced Biomedical Sciences, University of Naples "Federico II", Naples, Italy.

[2]Department of Clinical Medicine and Surgery, University of Naples "Federico II", Naples, Italy.

[+] These authors contributed equally to this work

***CORRESPONDENCE:** Prof. Silvana Del Vecchio, Via Sergio Pansini 5, 80131 Naples, Italy; phone +390817463307; Fax: +390815457081, e-mail: delvecc@unina.it

Supplementary Table S1. Conventional and texture features derived from [^{18}F]FDG PET/CT images by using LIFEx software

FEATURES			
SHAPE	HISTOGRAM	DERIVED FROM CONVENTIONAL	CONVENTIONAL
SHAPE Sphericity<br>SHAPE Compacity<br>SHAPE Volume	HISTO_ Skewness<br>HISTO_Kurtosis<br>HISTO_Entropy-log_{10}<br>HISTO_Energy	CoV (SD/SUVmean)	SUVmin<br>SUVmax<br>SUVmean<br>SD mean<br>SUVpeak<br>MTV<br>TLG
GLCM	GLRM	NGLDM	GLZLM
GLCM_Homogeneity<br>GLCM_Energy<br>GLCM_Contrast<br>GLCM_Correlation<br>GLCM_Entropy-log_{10}<br>GLCM_Dissimilarity	GLRM_SRE<br>GLRM_LRE<br>GLRM_LGRE<br>GLRM_HGRE<br>GLRM_SRLGE<br>GLRM_SRHGE<br>GLRM_LRLGE<br>GLRM_LRHGE<br>GLRM_GLNU<br>GLRM_RLNU<br>GLRM_RP	NGLDM_Coarseness<br>NGLDM_Contrast<br>NGLDM_Busyness	GLZLM_SZE<br>GLZLM_LZE<br>GLZLM_LGZE<br>GLZLM_HGZE<br>GLZLM_SZLGE<br>GLZLM_SZHGE<br>GLZLM_LZLGE<br>GLZLM_LZHGE<br>GLZLM_GLNU<br>GLZLM_ZLNU<br>GLZLM_ZP

Supplementary Table S2. Accuracy of models using SVM method in the training dataset

	Number of combined features				
Lasso method	First feature	Two first features	Three first features	Four first features	Five first features
**Fscchi2**	81.1	78.3	81.1	83.7	83.7
**Fscmrmr**	75	73	75	71.4	78
**Fscnca**	75	73.1	78.4	79	-
**Fsrftest**	81.1	78.3	81.1	83.7	83.7
**Fsrnca**	75	73.1	78.4	79	-
**Fsulaplacian**	77.2	74	79	83.7	81.1
**Relieff**	75	72.1	80.5	78	83.7

Supplementary Table S3. Accuracy of models using SVM method in the final testing dataset

	Number of combined features				
Lasso method	First feature	Two first features	Three first features	Four first features	Five first features
**Fscchi2**	48	46.4	50.5	45	51.3
**Fscmrmr**	40	47	42	49	47
**Fscnca**	49	45.3	44	50.9	-
**Fsrftest**	48	46.4	50.5	45	51.3
**Fsrnca**	49	45.3	44	50.9	-
**Fsulaplacian**	49	43	47.2	49.3	50.9
**Relieff**	47	49	45.3	51	49.4
